# Frequency of extreme precipitation increases extensively with event rareness under global warming

**DOI:** 10.1038/s41598-019-52277-4

**Published:** 2019-11-05

**Authors:** G. Myhre, K. Alterskjær, C. W. Stjern, Ø. Hodnebrog, L. Marelle, B. H. Samset, J. Sillmann, N. Schaller, E. Fischer, M. Schulz, A. Stohl

**Affiliations:** 1 CICERO Center for International Climate Research – Oslo, 0318 Oslo, Norway; 2 Institute for Atmospheric and Climate Science, ETH Zurich, 8092 Zurich, Switzerland; 3 Norwegian Meteorological Institute, 0313 Oslo, Norway; 4 NILU – Norwegian Institute for Air Research, Kjeller, Norway

**Keywords:** Climate sciences, Environmental social sciences

## Abstract

The intensity of the heaviest extreme precipitation events is known to increase with global warming. How often such events occur in a warmer world is however less well established, and the combined effect of changes in frequency and intensity on the total amount of rain falling as extreme precipitation is much less explored, in spite of potentially large societal impacts. Here, we employ observations and climate model simulations to document strong increases in the frequencies of extreme precipitation events occurring on decadal timescales. Based on observations we find that the total precipitation from these intense events almost doubles per degree of warming, mainly due to changes in frequency, while the intensity changes are relatively weak, in accordance to previous studies. This shift towards stronger total precipitation from extreme events is seen in observations and climate models, and increases with the strength – and hence the rareness – of the event. Based on these results, we project that if historical trends continue, the most intense precipitation events observed today are likely to almost double in occurrence for each degree of further global warming. Changes to extreme precipitation of this magnitude are dramatically stronger than the more widely communicated changes to global mean precipitation.

## Introduction

It is well established that the intensity of extreme precipitation increases more strongly with global mean surface temperature than mean precipitation^1–5^, as the latter, on a global scale, is limited by energy constraints^6–9^. While a full scientific understanding of the processes that link heavy precipitation events to global warming is still lacking, recent literature includes a number of advances^3,4,10–13^. Globally, the observed intensity in daily heavy precipitation events, i.e. the rainfall per unit time, increases with surface temperature at a rate similar to that of vapour pressure (6–7% K^−1^)^4,6,14,15^. Also, simulations by recent Earth System Models produce changes in the annual maximum precipitation intensity that are relatively similar to observations, with a small low bias^16,17^.

The increase in the frequency of extreme precipitation, i.e. the number of events per unit time with intensity above a given threshold, has generally received much less attention^18–21^. Unlike for intensity changes, the Intergovernmental Panel on Climate Change (IPCC) Fifth Assessment Report (AR5) gave no quantitative estimates of frequency changes^22^. Recent analyses of observations over Europe^4,23^ and over the US^23–26^ however show a substantial frequency increase. Here, we analyse a comprehensive data set of changes in the total amount of water falling as extreme precipitation, quantifying the contributions from changes in the intensity and the frequency, and including both observed and simulated precipitation. We investigate events that are rarer than those used in earlier studies, and find larger changes in the total amount of extreme precipitation than has been previously quantified.

**Figure 1:**
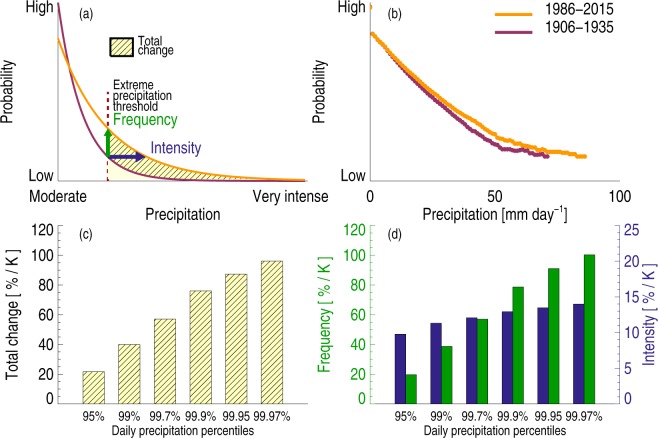
Schematic illustration of the probability density function (PDF) of daily precipitation amount (**a**). The purple line shows a reference PDF, and the orange line shows how it changes with higher temperatures. For a certain precipitation amount (vertical dotted line), the PDF shift can be decomposed into an increase in intensity (illustrated by the horizontal blue arrow) and an increase in frequency (vertical green arrow). The increase in the total extreme precipitation above a certain threshold is illustrated by the shaded yellow area, which combines intensity and frequency increases. The actual PDFs for two time periods 1906 to 1935 and 1986 to 2015 for the mean of 15 rain gauge stations in the Netherlands (**b**). Total extreme precipitation changes from E-OBS data between the two periods 1951–1980 and 1984–2013 (**c**) and the frequency and intensity contribution to total extreme precipitation change (**d**) for daily precipitation percentiles and scaled by global and annual mean temperature change to derive units of %/K. The 95^th^ percentile occurs on average once in 20 days, 99^th^ percentile once in 100 days, 99.7^th^ percentile once in 333 days, 99.9^th^ percentile once in 1000 days, 99.95^th^ percentile once in 2000 days and 99.97^th^ percentile once in 3333 days.

To illustrate how changes to the total extreme precipitation are affected by both frequency and intensity, Fig. 1a shows a conceptualized probability density function (PDF) of daily precipitation corresponding to a reference surface air temperature (purple line), compared to one with a higher surface air temperature (orange). The increase in the intensity of heavy precipitation is illustrated by the horizontal blue arrow; the increase in frequency as the vertical green arrow. If we define “extreme” precipitation to be any event above a certain percentile, as illustrated by the dotted vertical line, Fig. 1a demonstrates that the total change in extreme precipitation amounts depends on changes to both intensity and frequency.

The features of Fig. 1a can also be seen in observations. Figure 1b shows a mean of measurements from 15 stations over the Netherlands covering more than 100 years of data (Methods). The observed maximum daily precipitation intensity increases by about 20 mm/day between the two 30-year periods analysed, while the frequency of precipitation events above any given threshold for “heavy” events also increases.

A common way to analyse changes in extreme precipitation is to follow the evolution of the percentiles of the daily precipitation PDFs^4,18,27,28^. Figure 1c shows the increase in precipitation (between two 30-year periods) over Europe for extreme events ranging from percentiles of 95% (occurring on average once every 20 days) via 99.9% (occurring once every 1000 days) to 99.97% (occurring on average about once every 10 years). Figure 1d shows that the strong monotonic increase in the change of total extreme precipitation with rareness of events arises mainly from the strong enhancement in the frequency.

**Figure 2:**
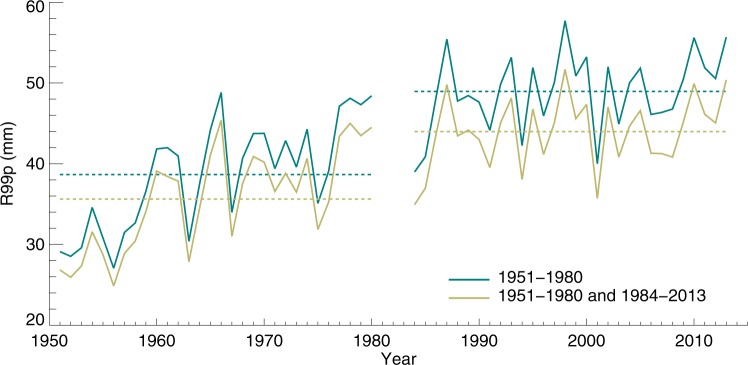
R99p over Europe from the E-OBS dataset. The green line uses 1951–1980 as a reference period at each grid point for the threshold of the 99th percentile of daily precipitation and this is applied for the 1984–2013 period, which is the standard approach in this study. The yellow line uses first the whole 60-year period (1951–1980 plus 1984–2013) for the calculation of the 99th percentile of daily precipitation on a grid point level and then how the mean varies over this 60-year period. The dotted lines show the mean of R99p for the two periods (1951–1980 and 1984–2013).

In this study, we investigate extreme precipitation events through established indices, such as the amount of daily precipitation above the 99^th^ percentile (R99p)^29^, which is equivalent to the total precipitation falling during the 1% heaviest precipitation events. Additionally, we define a number of extended indices that quantify even more extreme events than R99p. Figure 2 shows the change in R99p over Europe between two selected time periods. The daily precipitation percentiles are calculated from the 30-year reference period (green line). Note that our conclusions are robust against perturbations to this reference period (Fig. 2, green vs. yellow line).

The R99p index considers only the wet days (daily precipitation greater than 1 mm), while recent literature recommended analysing all days of the year, independent of precipitation amount^28^, to account also for changes in the number of precipitating days. When investigating the heaviest precipitation events (occurring seldom) we therefore create indices for R99.95p_all_ and R99.97p_all_, and include all days in the analysis. Supplementary Figure S1 shows the relationship between our various indices, for wet days and all days over Europe. From these defined indices, we can quantify the total precipitation change above a certain percentile (e.g. 99^th^ or 99.97^th^ percentiles) and then go on to investigate the contributions from changes in frequency and intensity separately. The frequency change is simply the change in number of events with precipitation above the fixed threshold obtained for the reference period for a given percentile, while the change in intensity is calculated from the precipitation amount above the percentile in question (see Methods). In addition, for consistency with other studies, we include the common climate index Rx1day. Rx1day is defined as the annual maximum daily precipitation, and is close to the 99.7^th^ percentile applied for all days. The spatial pattern of Rx1day change shows an overall increase over all continents over the historical era^16^ and has been investigated in several earlier studies^16,30^.

We use measurements from individual meteorological stations over some selected regions, as well as gridded precipitation products over Europe, USA, Japan and Australia. These regions were chosen based on the availability of high quality long term data. The observations are analysed for one recent and one reference period of approximately 30 years using time series going backward in time as far as allowed by observational records (see Methods). In addition to observational data, we use results from several global coupled atmosphere-ocean general circulation models (GCMs) from the Coupled Model Intercomparison Project Phase 5 (CMIP5)^31^. The model results are analysed over the historical period and for a future scenario with high greenhouse gas emissions (RCP8.5)^32^. Changes in precipitation are consistently reported as relative change divided by global mean surface temperature change between the selected time periods. A further description of the methodology is given in Methods.

## Results

**Figure 3:**
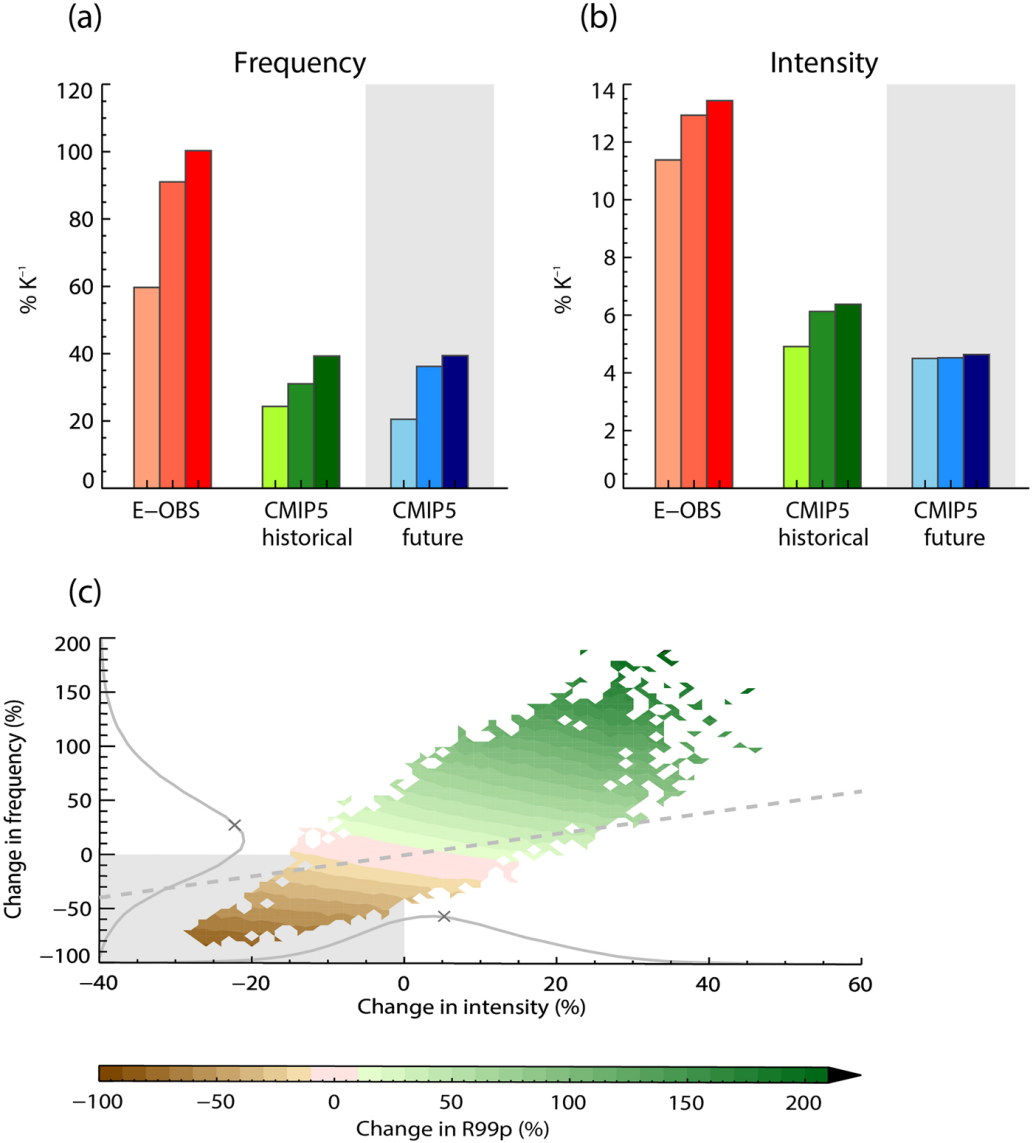
Changes in frequency (**a**) and intensity (**b**) of extreme rainfall events in observations (E-OBS) and models (CMIP5) between the two periods 1951–1980 and 1984–2013 over Europe. Historical and future (1984–2013 versus 2071–2100) model simulations are shown. The lightest colour is for changes contributing to R99p, medium colour to R99.95_all_ and darkest colour for contributions to R99.97_all_. Total extreme precipitation change (R99p) shown as a function of change in intensity and frequency for observational data (E-OBS) over Europe (**c**) for time periods 1951–1980 versus 1984–2013. The colour scale refers to percentage change in R99p. PDFs of intensity and frequency changes are shown on the x-axis and y-axis, respectively. Crosses on the PDFs are mean values of the PDFs and scale to the results in (**a**,**b**) for R99p if one accounts for a temperature change of 0.46 K.

Figure 3a,b show how the frequency and intensity of extreme precipitation events increase with global warming over Europe, for three of the indices described above. We find a strong observed increase in extreme precipitation frequency per degree warming, and with a magnitude that increases monotonically with the “extremeness” of the chosen threshold percentile – or, equivalently, with the rareness of the event. For instance, for R99p, the observed changes in frequency and intensity are 60% K^−1^ and 11% K^−1^, respectively, but reach 100% K^−1^ and 13% K^−1^ for R99.97p_all_. Observed increases in frequency and intensity are generally larger over Europe and Japan than over USA and Australia (see Supplementary Figure S2). Furthermore, Supplementary Figure S1 shows that results using the standard definition of R99p, including only wet days, differ from extreme indices using all days. This is consistent with our results, since 1% of all days is a less strict selection threshold compared to 1% of wet days. The increase in frequency is also consistent with previous results using the same observational data set over Europe^4^ given that our results are normalized by temperature change and shown here at more extreme percentiles. Similarly, the increase in intensity globally over land is consistent with earlier findings^2,16^.

**Figure 4:**
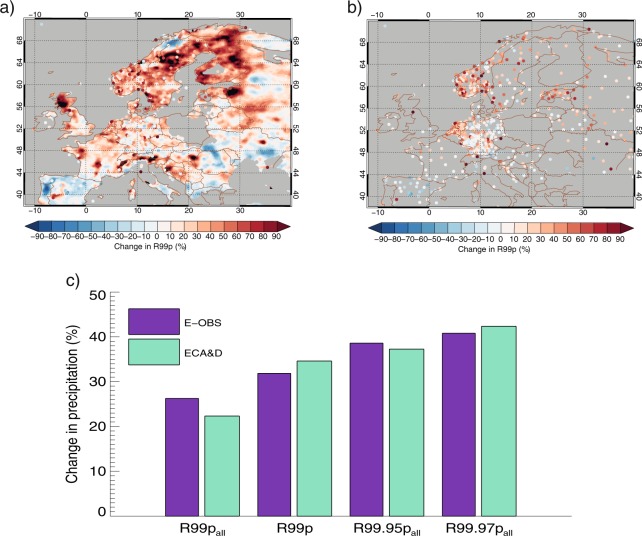
Change in R99p in E-OBS (**a**) and precipitation station data part of the ECA&D database (**b**) between the two periods 1951–1980 and 1984–2013 (same as in Fig. 1 and Fig S1). E-OBS is plotted as a contour plot, while the colored dots represent precipitation station results of change in R99p. Here, when several gauge data stations are available within an E-OBS grid point, we have averaged their measurements. The requirement of including stations is at least 80%-time coverage. Comparison of E-OBS and ECA&D for the extreme precipitation indices used in this study show good agreement, with no systematic bias between the two data sets (**c**).

Gridded datasets have the limitation that the number of stations affecting each grid point may change over time. In addition, averaging extremes within a grid point may reduce any high signals of change. We have investigated this by using measurements from the gauge data (ECA&D – European Climate Assessment & Dataset) and the gridded products (E-OBS) where these are comparable, see Fig. 4. Figure 4c shows good agreement between regional mean E-OBS and ECA&D where these can be compared of the extreme precipitation indices used in this study (within 20% for R99p_all_ and within 5% for the other indices), with no systematic bias between the two data sets.

Figure 3c shows that the change in total extreme precipitation is a combination of increases in the intensity and frequency but is dominated by the frequency change as shown by the PDFs. However, the intensity increase is clearly also of importance. For one third of the grid points, the change in the total amount of rainfall in extreme events (R99p) is at least 20% greater than expected from changes in frequency alone. The global climate models also show strong increases in the intensity and frequency for the considered extreme indices (Fig. 3a,b) but these increases are smaller than in the observations, which is consistent with earlier findings^16,17^. However, Supplementary Figure S3 shows that a subset of the CMIP5 models simulate changes that are as large as in the observations. Historical and future changes are consistent in the climate models over Europe, but are somewhat larger in the future when including USA, Japan and Australia. Supplementary Figure S4 shows that the change in the frequency of extreme precipitation for a five-day period is of a similar magnitude as for one day for the rarest events, but about half of the strengthening for R99p (Fig. 3a vs Supplementary Figure 4a).

When discussing very rare events, regional intrinsic variability is naturally a limiting factor. By using climate model data from the pre-industrial period, and comparing randomly selected time periods, we have investigated what differences in extreme value indices could be expected due to natural variability, i.e. with no forced changes in surface temperature in response to climate drivers such as greenhouse gases and aerosols. We find that changes in R99p and R99.97p_all_ between randomly selected periods are small compared to the findings in this study from historical and future simulations. A mean of four climate models over three different 30-year time periods gave 0.8% ± 3.1% and 1.6% ± 7.9% changes for R99p and R99.97p_all_, respectively, where the uncertainty range is given as one standard deviation.

**Figure 5:**
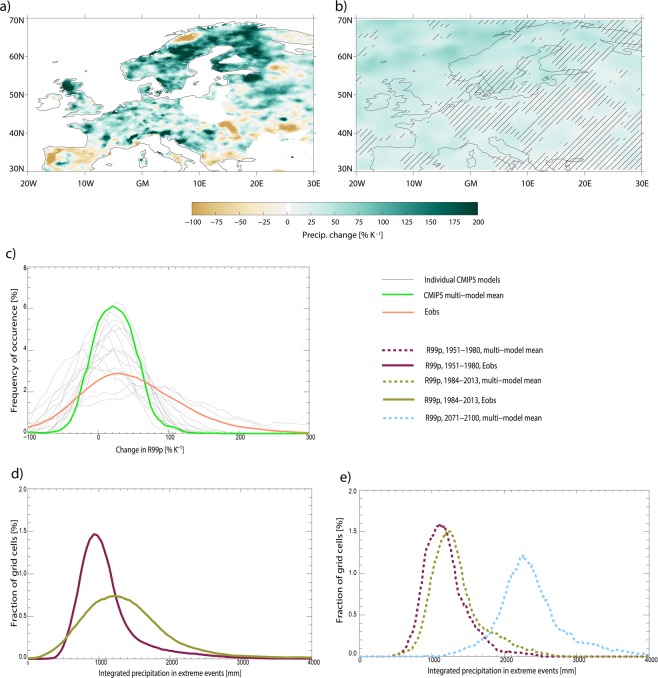
Regional distribution of the change in R99p over Europe from E-OBS between the two periods 1951–1980 and 1984–2013 (**a**), mean of 16 CMIP5 models 1951–1980 and 1984–2013 (**b**), PDFs of 16 individual CMIP5 models, their model-mean PDF and observations (**c**), PDF of observed R99p for two time periods (**d**), PDF of model simulated R99p for historical, present, and future (2071–2100) (**e**). Note the x-axis in panel (**d**,**e**) refers to the sum of R99p over 30-year periods. In panel b, hatching is provided for grid cells where more than 4 of the 16 models disagree on the sign of the change.

As seen in Fig. 3c, the change in rainfall intensity, change in event frequency and the total change in heavy precipitation amounts, are all negative in certain parts of Europe (R99p is negative for about 25% of all grid points, see also the geographical pattern in Fig. 5a). Negative values due to natural variability can be expected to be partly cancelled by an equal amount of positive values and the domain average results shown in Fig. 3a will then cancel out this effect. To illustrate that the negative values are partly due to short observational time series, and therefore strongly influenced by natural variability on a local scale, in Fig. 5 we use results from 16 free running CMIP5 climate models for the same period as used in Fig. 3. Each of the models shows a similarly patchy pattern in the change of the total extreme precipitation as the observations (Supplementary Figure S5). However, the mean of the 16 models (Fig. 5b) shows a more robust estimate of the underlying forced response and thus a rather homogeneous pattern relative to the individual models. Part of the changes in southern-Europe may be influenced by changes in dynamics^11^. Illustrating the precipitation changes as PDFs, the observed changes have a width and shape similar to the individual models, but the PDF of the multi-model mean is narrower (Fig. 5c). Using even longer time series of 50 years further increases the homogeneity of the multi-model mean changes (as shown for future changes in Supplementary Figures S6 and S7), but large inhomogeneous patterns can still be seen for each of the individual models. In the future, extreme precipitation will still be heavily influenced by local natural variability, so that at individual stations or in smaller regions the signal will be small compared to the variability. Therefore, it is necessary to aggregate measurements from larger regions in order to detect changes in extreme precipitation, as done earlier^4^ and in the present study, or to use statistical methods for deriving long-term trends^33^. It is particularly worth noticing that only a few of the models capture the very strong observed changes (more than 100% K^−1^) as shown in Fig. 5c.

Figure 5d,e show a shift in the PDF of R99p over Europe both within the observed record and between historical and future CMIP5 simulations. The shift of the PDF is particularly strong for the most extreme events taking place in regions with historically high R99p, whereas for the observations there is a small reduction in R99p in regions of low extreme precipitation. In the observations, the PDF therefore widens for the recent period compared to the reference period, whereas for the multi-model mean the PDFs mostly have a more similar shape. The future R99p changes under the high greenhouse gas emission scenario are much larger than in the historical simulations, consistent with their much larger surface temperature changes. Overall, the shift in the PDF towards higher values of total precipitation from extreme events is clearly seen in both observations and climate models.

**Figure 6:**
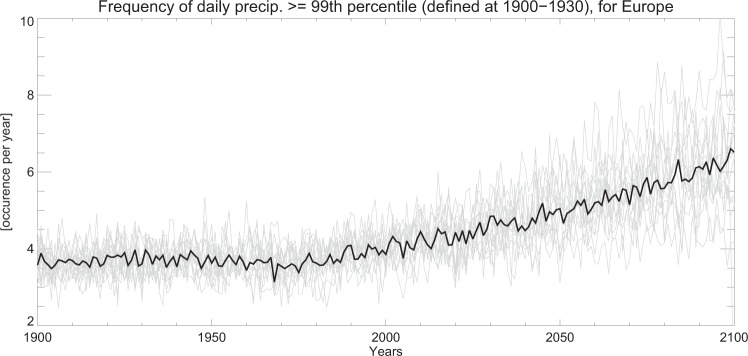
European regional mean frequency of daily 99^th^ percentile of precipitation simulated by CMIP5 models for historical and future conditions (1900–2100). The percentile is calculated in a reference period covering 1900 to 1930. The thin grey lines are for individual CMIP5 models and the solid line is for the multi-model mean.

Figure 6 shows the mean frequency of precipitation events above the 99^th^ percentile over Europe, for historical and future simulations (1900–2100), including individual CMIP5 models and the multi-model mean. Here, 1900–1930 is used as a reference period. Per definition the mean frequency during 1900–1930 for R99p_all_ is 3.65 (1% of the days in a year) with the models showing some interannual variability with a regional mean from 2 to 5 events per year. The model diversity in precipitation frequency is large during the 21^st^ century, with a multi-model mean around 6 events at the end of the century. A major driver of model diversity in the change in frequency between the end of the century and 1900–1930, is the fact that models have different surface temperature changes dominated by differences in the climate sensitivities. Models that have the strongest (weakest) increase in frequency also have the strongest (weakest) surface temperature increase. The change in frequency follows the change in surface temperature between various 30-year periods to within 1% for the multi model mean (not shown).

**Figure 7:**
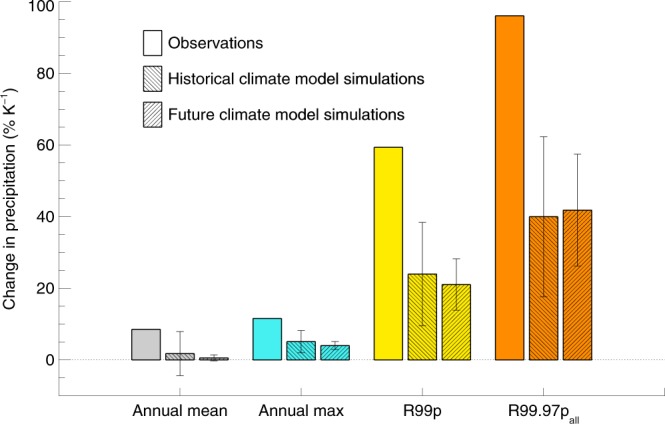
Change in temporal mean precipitation, annual maximum precipitation (Rx1day), R99p, and in R99.97p_all_ in observations (1951–1980 versus 1984–2013) and CMIP5 climate models (1951–1980 versus 1984–2013 for the historical period and 1984–2013 versus 2071–2100 for future) over Europe. Whiskers around model averages give the spread between individual model results as plus minus one standard deviation.

Figure 7 shows the changes in mean and annual maximum (Rx1day) precipitation intensities over Europe. These results are generally consistent with the assessments in IPCC AR5^2^, but with stronger mean observed changes. However, the increase in total precipitation from extreme events such as the 1% heaviest rainfall (R99p, yellow) and events occurring on average about once per decade (R99.97p_all,_ orange) in the control period, is several times larger than the intensity increase discussed in IPCC AR5. These increases reach 59% K^−1^ and 96% K^−1^, respectively, in the observations, which is five and ten times larger than the intensity increases alone. The observed changes are about twice as large over Europe and Japan than over the USA and Australia (see Supplementary Table S1). Global historical and future climate models give a smaller increase than the observations for R99p and R99.97_all_ over Europe (Fig. 7) and over other land areas (Supplementary Table S2). Whether the stronger observed increase over Europe and Japan is driven by large natural variability, different regimes for extreme precipitation, artefacts in the measurements or other factors are unclear. However, the mean of the global models also shows stronger historical changes in extreme precipitation over Europe and Japan compared to USA.

## Discussions

Global warming increases both the intensity and frequency of extreme precipitation, so to characterize the full response of extreme precipitation to global warming, either their total or both of their individual contributions must be communicated. The duration^34^ and area^35–37^ of the extreme precipitation events may also be affected by climate change. We have shown here that extreme precipitation events occurring on average twice per decade will increase in frequency by 1–2 events per decade per degree of warming. Thus, for a 2 K global mean surface warming, the frequency of these events would double or triple. Further, observations indicate that the total precipitation from extreme events occurring once per decade may increase on the order of 10 times more than when considering intensity increases alone. Changes in all aspects of heavy precipitation are vital to society, for instance when it comes to constructing sufficiently resilient infrastructure. Traditionally, infrastructure design is based on Intensity-Duration-Frequency (IDF) curves but they assume a constant climate^38^. Unsurprisingly, this has been shown to underestimate the effect of extreme precipitation on infrastructure^39^. There is therefore a strong need to update infrastructure design strategies in a way that climate change is taken into account, especially since changes in intensity and frequency of precipitation extremes have already been detected in some regions^40^, meaning that existing infrastructure is already ill-adapted. Updated IDF curves for the US using CMIP5 simulations show similar results, i.e. a 20% increase in intense precipitation events that occur twice as often in some highly populated regions^41^ and recently the importance of changes in temporal patterns of heavy precipitation events on urban and suburban flooding has been highlighted^42^. Changes in the frequency of extreme precipitation events is therefore highly relevant, especially where the resilience of existing infrastructure is already exceeded.

Here we highlight the fact that total changes in extreme precipitation are a result of combined changes in both intensity and frequency. A main result from this study is that there is a strong monotonic strengthening of frequency increase and thereby relative increase in total extreme precipitation with rareness of the precipitation events. We argue that the impact of extreme precipitation on society results from both the number of events above the present tolerance and the severity of these events, and hence indices used to indicate impacts of climate change should encompass both. These increases are much higher than found in studies of precipitation intensity alone^1,43^, including the IPCC 5^th^ Assessment Report. Such large increases are not taken into account by adaptation management, and our findings imply that society may not be adequately prepared for the coming changes in extreme rainfall.

## Methods

### Calculation of extreme indices

The extreme precipitation indices are calculated as follows. For R99p, we consider wet days only and find the 99^th^ percentile of daily precipitation events in the reference period for each individual observational or model grid point. This threshold is then used for both the reference and the latest period and R99p is calculated as the sum of the precipitation during all events exceeding the threshold. We then area-average the results from each period and calculate the change in R99p for the given region. In this study we use the first 30-year period as a reference period and find the percentiles from these data. This choice of reference period has little effect on the resulting change in R99p, as can be seen in Fig. 2. For R99.95p_all_ and R99.97p_all_, the same method is applied, except all days (not only days with precipitation above 1 mm) are included when calculating the reference period 99.95^th^ and 99.97^th^ percentiles. These latter precipitation indices provide more extreme events compared to R99p and they occur on average only about every fifth and tenth year, respectively. The mathematical expression for R99p is given at the following web site along with 26 other key climate extreme indices (http://etccdi.pacificclimate.org/list_27_indices.shtml) and the historical development of these climate extreme indices have been described^29^. R99.97p_all_ has the same expression as R99p except that the summation is over all days instead of only wet days and the 99.97^th^ percentile is applied instead of the 99^th^ percentile. Figure S1 compares climate indices for wet and all days. For R99.97_all_ it will be 4 events at every location for the reference period and a change in total precipitation can be either due to the stronger intensity, increase in the frequency or a combination of the two. The frequency change is calculated as the change in number of precipitation events above the threshold given by the reference period. The change in intensity is calculated as the total precipitation in the events above the percentile in the reference period and the strongest equal number of events in the recent/future period. Note that the change in total, intensity and frequency are calculated independently. To scale the observed changes we use global and annual mean temperatures from NASA GISS data (https://data.giss.nasa.gov/gistemp/)^44,45^ to derive units of %/K.

### Observational data

There are several limitations in the measurement data available for extreme precipitation analysis^29^. First of all, long term measurements are only available for some land areas; secondly there are several issues related to quality control^29^. To limit the influence of these issues on our results, we have chosen to focus our analyses on regions for which long and continuous time series exist and to combine this with climate model simulations.

This study makes use of the following gridded observational datasets of precipitation. For Europe, version 15 of the E-OBS dataset is used for the periods 1951–1980 and 1984–2013^46^. It covers the land regions bound by 25N to 75N and 40W to 75E on a 0.25-degree resolution. For the USA, the CPC (Climate Prediction Center) Unified Gauge-Based Analysis of Daily Precipitation is used for the periods 1948–1976 and 1978–2006. This dataset covers the contiguous United States on a 0.25-degree resolution^47^. For Japan, version 1207 of the APHRODITE (Asian Precipitation - Highly-Resolved Observational Data Integration Towards Evaluation) Japan Precipitation dataset is used for 1900–1929 and 1982–2011^48^. It covers the islands of Japan on a 0.05-degree resolution. We also make use of the Australian Daily Rainfall Gridded Data for the periods 1951–1980 and 1984–2013 on a 0.05-degree resolution^49^. Here, we only consider grid points over land for which the fraction of days with rain is a minimum of 2%. This is to avoid unclear results from data sparse desert regions. For all gridded datasets, we require each grid point to have continuous data throughout both our periods of interest. We have further used observational data from rain gauges found in the non-blend version of the European Climate Assessment’s daily dataset of 20^th^-century surface air temperature and precipitation including data in Fig. 1^50^.

### Climate model data

In the analysis we used precipitation data from 16 coupled climate models contributing to the Coupled Model Intercomparison Project Phase 5 (CMIP5)^31^ for the historical (1850–2005) and one future scenario RCP8.5^51^. Supplementary Table S3 shows that the frequency change is increasing relatively linearly with temperature in the CMIP5 models. Names of the individual models are given in Supplementary Figs S6 and S7. Multi-model mean is the average across the post-processed model results. In normalising the precipitation change by temperature for each of the model, the global mean temperature for the same model is used to derive changes of units %/K.

### Additional information

No competing non-financial interests, but one of the research projects funding this work has received a small part (less than 3% of the total budget) of the funding from an insurance company, If.

## Supplementary information

Supplementary material

## Data Availability

The E-OBS dataset from the EU-FP6 project ENSEMBLES (http://ensembles-eu.metoffice.com) and the data provided through the ECA&D project (http://www.ecad.eu) are publicly available. CPC US Unified Precipitation data is available by the NOAA/OAR/ESRL PSD, Boulder, Colorado, USA, from their Web site at https://www.esrl.noaa.gov/psd/ and are freely available. The DIAS APHRODITE dataset is archived and freely provided under the framework of the Data Integration and Analysis System (DIAS) funded by Ministry of Education, Culture, Sports, Science and Technology (MEXT). The Australian Daily Rainfall Gridded Data data is available through the Bureau of Meteorology. The CMIP5 data that support the findings of this study are openly available in the ESGF portal at http://esgf-node.llnl.gov/.
