# Supplementary material for: Frequency of extreme precipitation increases extensively with event rareness under global warming
Source: Sci Rep. 2019 Nov 5;9:16063. doi: 10.1038/s41598-019-52277-4 (PMC6831572; doi:10.1038/s41598-019-52277-4)
Supplement: Supplementary file 1 — Supplementary material [file 41598_2019_52277_MOESM1_ESM.pdf]

Supplementary material

Frequency of extreme precipitation increases extensively with event rareness under global warming

Myhre, G.[1*], K. Alterskjær[1], C.W. Stjern[1], Ø. Hodnebrog[1], L. Marelle[1], B.H. Samset[1], J. Sillmann[1], N. Schaller[1], E. Fischer[2], M. Schulz[3], A. Stohl[4]

[1]CICERO Center for International Climate Research – Oslo, 0318 Oslo, Norway
[2]Institute for Atmospheric and Climate Science, ETH Zurich, 8092 Zurich, Switzerland
[3]Norwegian Meteorological Institute, 0313 Oslo, Norway
[4]NILU – Norwegian Institute for Air Research, Kjeller, Norway

*corresponding author; gunnar.myhre@cicero.oslo.no

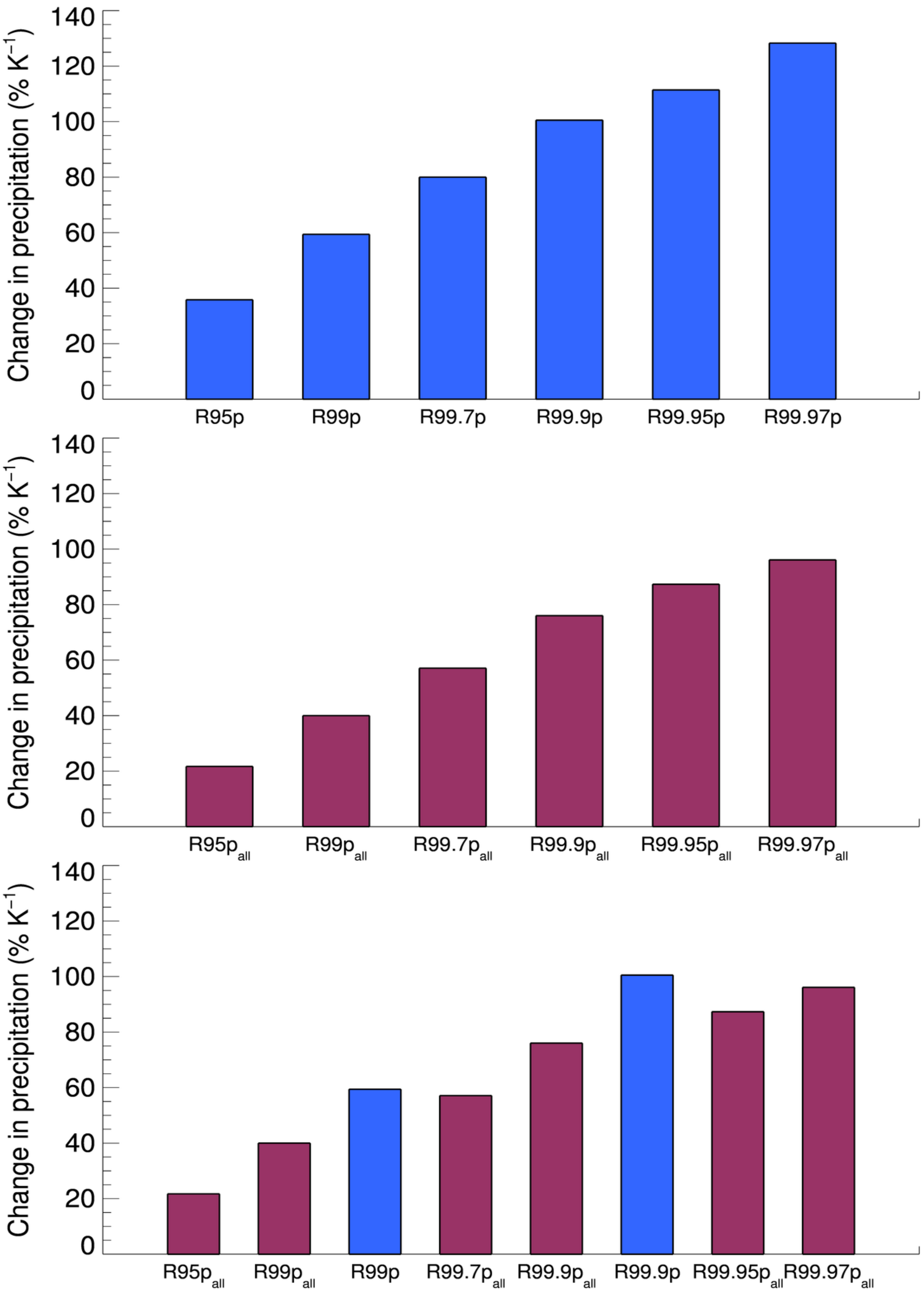

Fig. S1. Comparison of indices for wet days (upper), all days (middle) and a combination (lower) for E-OBS between the two periods 1951-1980 and 1984-2013.

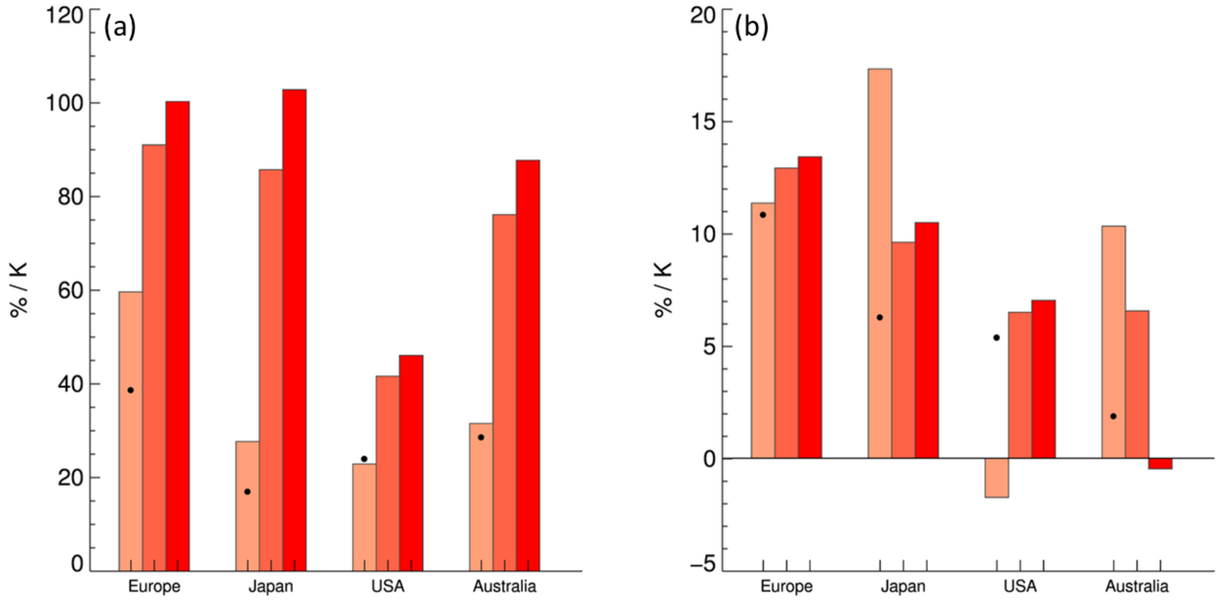

Fig. S2. Observed frequency (a) and intensity (b) changes given as percent change per degree warming (%/K) over Europe, Japan, USA, Australia (time periods given in Methods). The lightest color is for R99p, medium colour for $R99.95_{all}$ and darkest colour $R99.97_{all}$. The black dotted symbol for R99p is when using all days instead of only wet days. Change between two periods as described in the Methods are shown.

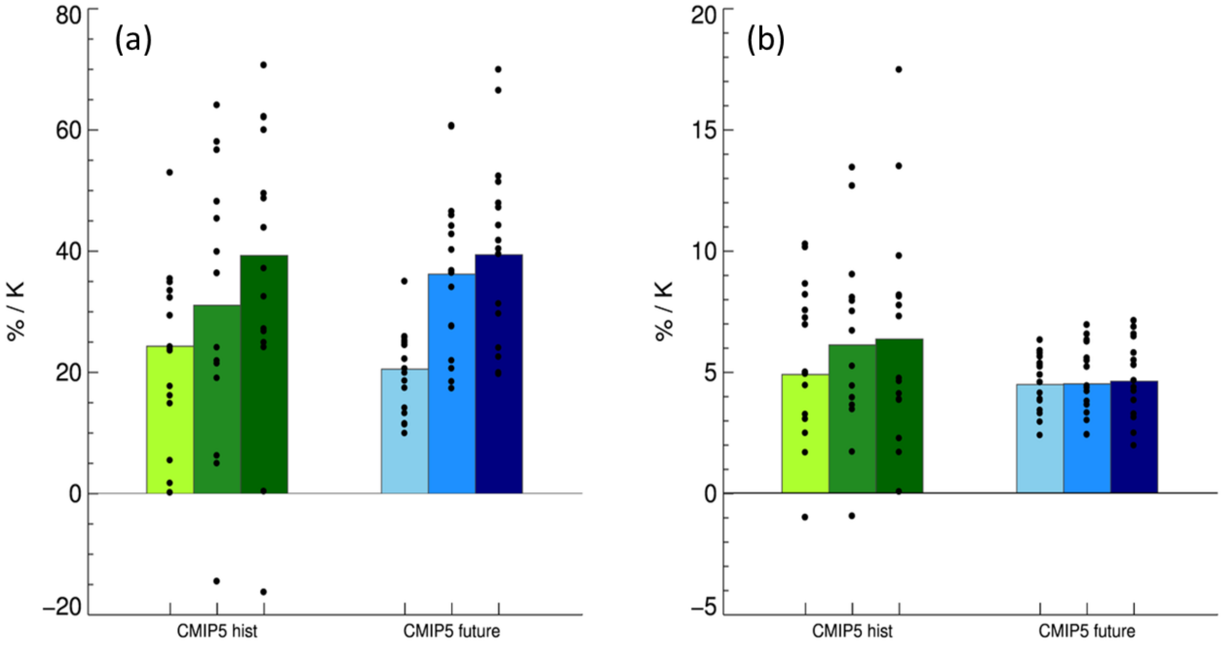

Fig S3. Changes in frequency (a) and intensity (b) in models given as percent change per degree warming (%/K) area weighted over Europe. Historical (between the two periods 1951-1980 and 1984-2013 and future (between 1984-2013 and 2071-2100) model simulations are shown. Median values shown in boxes and individual CMIP5 models with dots.

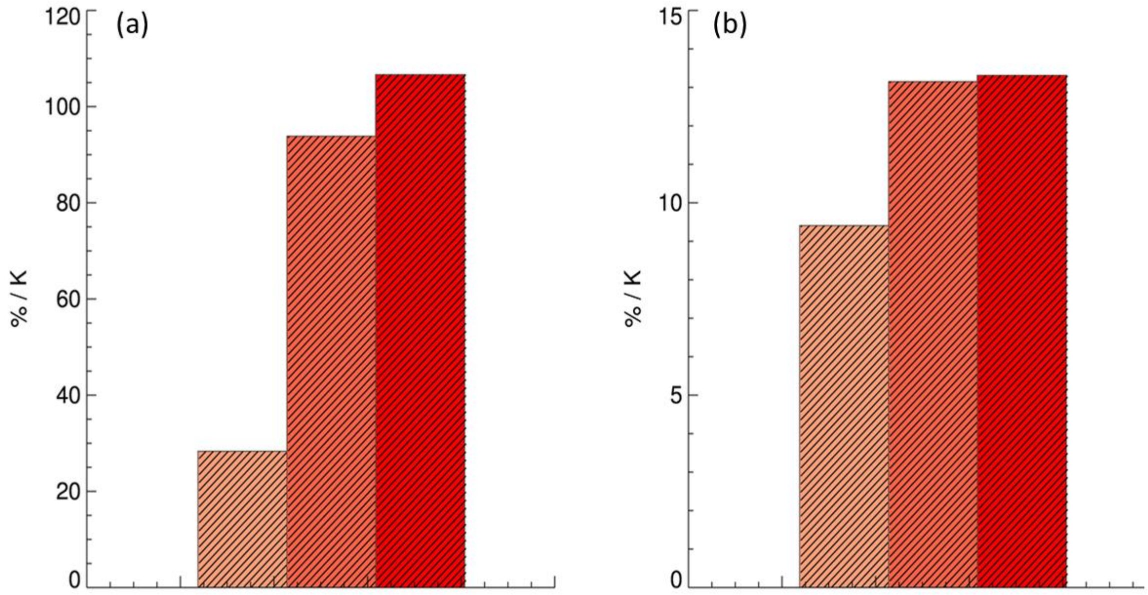

Fig S4. Observed (E-OBS) frequency (a) and intensity (b) changes given as percent change per degree warming (%/K) over Europe for five days precipitation (often described by the extreme index Rx5day). Here, the lightest color is for the 1% of the wettest days, medium colour for the 99.95th percentile and darkest colour 99.97th percentile, respectively R99p_{all}, R99.95$_{all}$, and R99.97p_{all}. The change is over the period 1951-1980 and 1984-2013.

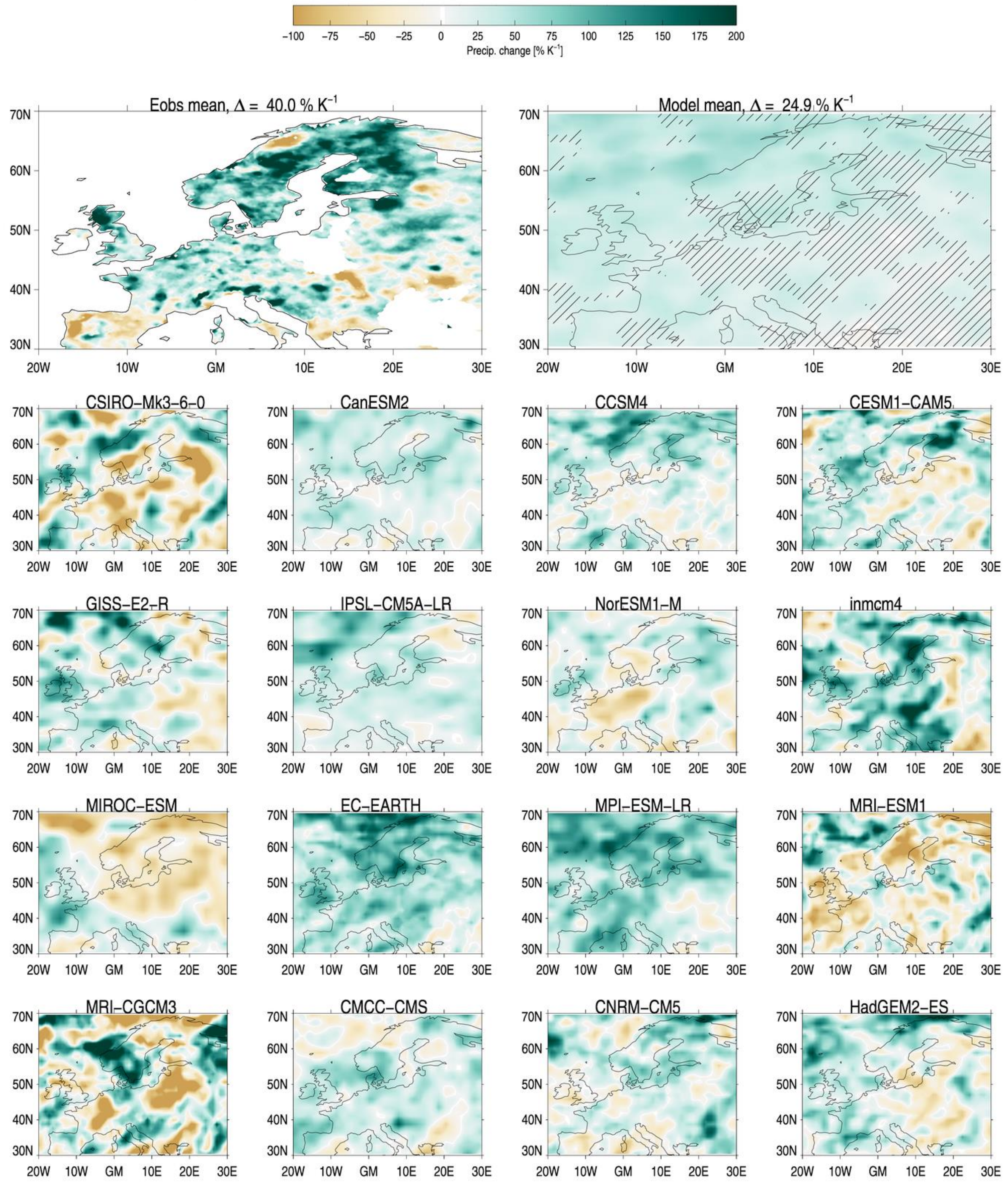

Fig S5. Regional distribution of change in R99p between the two periods 1951-1980 and 1984-2013 over Europe from observations (upper left), mean of 16 CMIP5 models (upper right) and 16 individual CMIP5 models. Hatching is provided for grid cells where more than 4 of the 16 models disagree on the sign of the change.

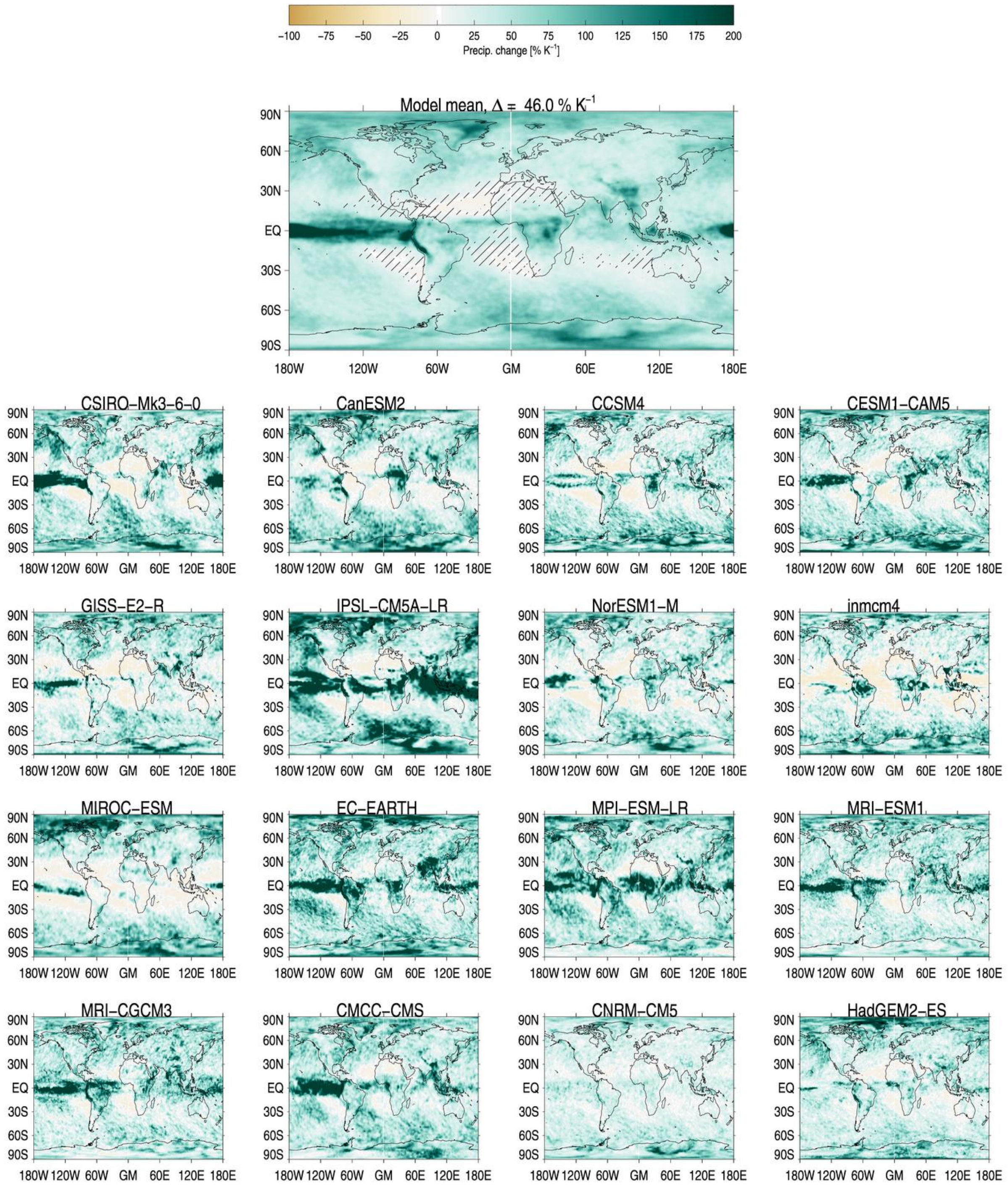

Fig S6. Global distribution of change in R99.97p_{all} over the 50-year period from 1851-1900 to 2051-2100 given as multi-model mean in the upper figure and for 16 individual CMIP5 models. Hatching is provided for grid cells where more than 4 of the 16 models disagree on the sign of the change.

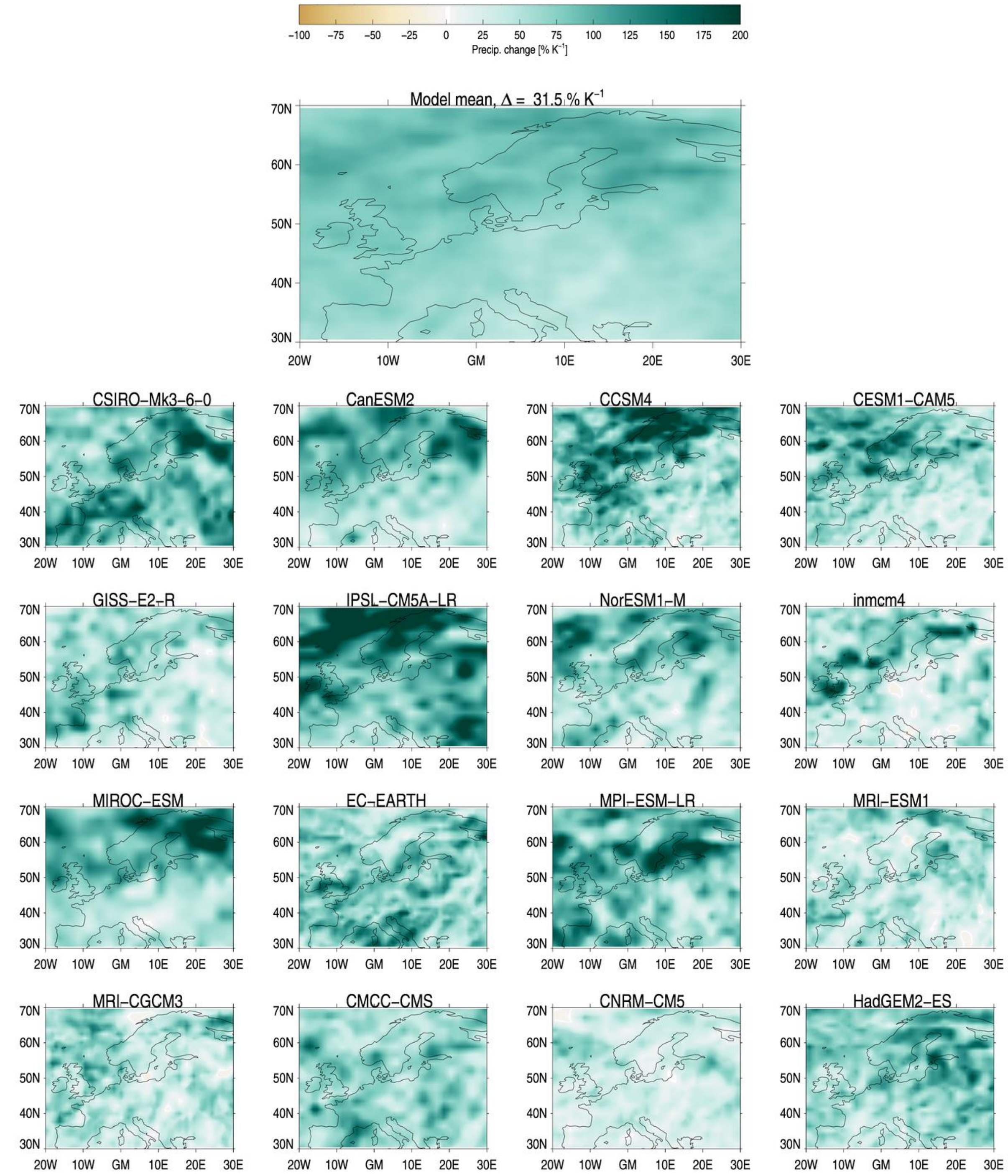

Fig S7. Regional change over Europe in R99.97p_{all} over the 50-year period from 1851-1900 to 2051-2100 given as multi-model mean in the upper figure and for 16 individual CMIP5 models.

Table S1.

Observed precipitation changes (% K^{-1}).

	Europe	USA	Japan	Australia	Area mean
Mean precipitation	8.5	16.2	-5.8	7.3	10.6
Rx1day	11.6	4.2	12.8	4.9	6.7
R99p	59.4	22.8	35.7	22.7	33.2
$R99.97p_{all}$	96.1	45.3	108.6	58.6	65.1

Table S2.

Multi-model change in historical and future extreme precipitation (%/K) for different land regions in 16 CMIP5 models.

**Extreme precipitation indices**	**Land historical**	**Land historical over same regions as observations**	**Land future**	**Land future over same regions as observations**
R99p	18.0	19.8	26.5	18.9
$R99.95p_{all}$	33.1	30.3	56.2	36.4
$R99.97p_{all}$	35.7	35.0	66.7	41.6

Table S3.

Multi-model mean change in frequency (%/K) for R99p and $R99.97_{all}$ for three time periods for 16 CMIP5 models.

**Period**[1]	**R99p**	**$R99.97_{all}$**
1851-1880 vs 1991-2020	18.73	51.01
1996-2005 vs 2011-2040	19.09	46.81
2006-2035 vs 2036-2065	18.14	46.78

[1]Temperature change for 1851-1880 vs 1991-2020 is 0.87K, 1996-2005 vs 2011-2040 is 0.91K and 2006-2035 vs 2036-2065 is 1.17K.
